# Secular Trends on Birth Parameters, Growth, and Pubertal Timing in Girls with Turner Syndrome

**DOI:** 10.3389/fendo.2018.00054

**Published:** 2018-02-28

**Authors:** Joachim Woelfle, Anders Lindberg, Ferah Aydin, Ken K. Ong, Cecilia Camacho-Hubner, Bettina Gohlke

**Affiliations:** ^1^Pediatric Endocrinology Division, Children’s Hospital, University of Bonn, Bonn, Germany; ^2^Endocrine Care, Pfizer Health AB, Sollentuna, Sweden; ^3^MRC Epidemiology Unit, Wellcome Trust-MRC Institute of Metabolic Science, University of Cambridge, Cambridge, United Kingdom; ^4^Endocrine Care, Pfizer Inc, New York, NY, United States

**Keywords:** Turner syndrome, height, growth, birth weight, birth length, puberty, secular trend

## Abstract

**Background:**

Whether children with chromosomal disorders of growth and puberty are affected by secular trends (STs) as observed in the general population remains unanswered, but this question has relevance for expectations of spontaneous development and treatment responses.

**Objectives:**

The aim of the study was to evaluate STs in birth parameters, growth, and pubertal development in girls with Turner syndrome (TS).

**Study design:**

Retrospective analysis of KIGS data (Pfizer International Growth Database). We included all TS patients who entered KIGS between 1987 and 2012 and were born from 1975 to 2004, who were prepubertal and growth treatment naïve at first entry (total number: 7,219). Pretreatment height and ages at the start of treatment were compared across 5-year birth year groups, with subgroup analyses stratified by induced or spontaneous puberty start.

**Results:**

We observed significant STs across the birth year groups for birth weight [+0.18 SD score (SDS), *p* < 0.001], pretreatment height at mean age 8 years (+0.73 SDS, *p* < 0.001), height at the start of growth hormone (GH) therapy (+0.38 SDS, *p* < 0.001) and start of puberty (+0.42 SDS, *p* < 0.001). Spontaneous puberty onset increased from 15 to 30% (*p* < 0.001). Mean age at the start of GH treatment decreased from 10.8 to 7.4 years (−3.4 years; *p* < 0.001), and substantial declines were seen in ages at onset of spontaneous and induced puberty (−2.0 years; *p* < 0.001) and menarche (−2.1 years; *p* < 0.001).

**Conclusion:**

Environmental changes leading to increased height and earlier and also more common, spontaneous puberty are applicable in TS as in normal girls. In addition, greater awareness for TS may underlie trends to earlier start of GH therapy and induction of puberty at a more physiological age.

## Introduction

Secular trends (STs) in birth parameters (1–3), growth (4, 5), and timing of puberty (6–8) are observed in normal populations in various settings. Changes in nutrition, better access to health care, and other environmental factors have been implicated as causative factors for these changes (4). Whether STs that affect normal populations also modulate growth and puberty of children with genetic or chromosomal disturbances that inherently affect growth and puberty remains unanswered.

Turner syndrome (TS) is caused by structural abnormalities in or complete loss of an X chromosome. It affects approximately 1 in 2,500 live-born female girls. The clinical phenotype of TS varies substantially, but in the majority of subjects includes short stature and ovarian failure, leading to hypogonadism and infertility (9).

In subjects with TS, haploinsufficiency of the SHOX gene has been proposed as an important cause of the growth phenotype in TS, since patients with heterozygous mutations in SHOX exhibit Leri-Weill syndrome, a bone dysplasia associated with short stature (10), whereas homozygous mutations with loss of both SHOX gene copies lead to a rare severe osteodysplasia (11). However, haploinsufficiency for the SHOX gene does not fully explain the growth phenotype and its variation in TS subjects. Probably, other factors such as estrogen deficiency (12), loss of additional X-chromosomal genes, or more general aneuploidy effects might be implicated in TS-associated short stature (13).

Although the majority of girls with TS have normal birth parameters, the frequency of TS in newborns with low birth weight (BW) and length is higher than expected (14, 15). This has been explained in part by loss or altered expression of X-chromosomal genes that are involved in fetal growth (16). Data analyzing the evolution of birth parameters in a sufficiently sized TS cohort over time to identify a ST in BW and length are lacking.

Girls with TS frequently exhibit delayed or absent pubertal development due to early ovarian failure. The exact molecular mechanisms leading to ovarian dysfunction in TS remain obscure. In a minority of patients (5–20%), puberty starts spontaneously and may even lead to spontaneous menarche in few subjects (17, 18). This seems to occur more frequently in subjects with a higher degree of mosaicism. As for auxological parameters, data on the presence or absence of an ST on spontaneous or induced puberty in TS are not available.

## Objectives

To assess STs on birth parameters, spontaneous growth and pubertal development in patients with TS and to evaluate whether clinical management of girls with TS has changed over time.

### Patients

The patients studied had received recombinant growth hormone (GH, Genotropin^®^, Pfizer Inc.) as part of the pharmacoepidemiologic survey known as KIGS^®^ (Pfizer International Growth Database). KIGS was established in 1987 as a worldwide observational registry to monitor outcomes and safety of Genotropin (somatropin, Pfizer Inc., New York, NY, USA) treatment in children with short stature. The KIGS survey was conducted in accordance with the Declaration of Helsinki (19).

As of June 2012, TS patients who entered the KIGS registry between 1987 and 2012 were included (total number: 7,219). Only patients who were at the prepubertal stage and naïve to any growth treatment at first entry were included. Birth years ranged from 1975 to 2004. The diagnosis of TS was made according to standard clinical practice and was confirmed by karyotype by the treating physicians. Patients’ characteristics are depicted in Table 1.

**Table 1:** Patients characteristics (total cohort).

Variables	*N*	Median	P10	P90	Mean	SD
Birth weight SD score (SDS)	6,372	−1.05	−2.52	0.39	−1.05	1.19
Birth length SDS	4,500	−0.87	−2.52	0.82	−0.85	1.41
Midparental Height SDS	6,571	−0.49	−2.01	1.00	−0.50	1.17
Height SDS at age 8 years (Prader)	1,091	−2.19	−3.23	−1.21	−2.20	0.81
Height SDS at age 8 years (Ranke)	1,091	0.13	−1.15	1.34	0.12	1.02
Age at the start of growth hormone (GH) tx	7,219	9.60	4.41	13.97	9.35	3.63
Height SDS at the start of GH tx (Prader)	7,219	−3.22	−4.55	−2.03	−3.26	1.06
Height SDS at the start of GH tx (Ranke)	7,132	0.19	−1.17	1.54	0.19	1.12
Weight SDS	7,219	−1.44	−3.06	0.30	−1.43	1.34
Body mass index SDS	7,218	0.31	−1.09	1.87	0.34	1.17
GH dose (mg/kg/week)	7,219	0.31	0.18	0.38	0.30	0.09

### Aims and Hypotheses

The main objective of this study was to assess STs on birth parameters, spontaneous growth, and pubertal development in patients with TS and to evaluate whether clinical management of girls with TS had changed over time. We had the following hypotheses:

– There is a positive ST for birth parameters.
– There is a positive ST for height before initiation of GH treatment.
– There is a positive ST for onset of puberty in the total TS cohort.

In order to address the outlined objectives, we analyzed data from three subcohorts derived from the KIGS database.

To assess STs, KIGS data on BW, birth length (BL), height SD score (SDS) at 8 years of age (chronological age 7.0–9.0 years) before initiation of any treatment, height SDS, and age at the start of GH therapy and at the start of puberty were analyzed in time intervals which were defined by year of birth (before 1980, 1980–1984, 1985–1989; 1990–1994; 1995–1999; and 2000–2004).

### Methods

Cohort 1a was used to assess trends both in pretreatment height and in the age at the start of GH therapy. Inclusion required sufficient pretreatment data without exposure to therapies affecting growth (GH, oxandrolone, and sex steroids). To assess trends in pretreatment height, TS girls born before 1980 were included for analyses regarding BL/BW and pretreatment height at 8 years (unless they started recombinant GH before 1985). To analyze trends in age at the start of GH therapy, TS girls born before 1980 were excluded (as the approval of TS as an indication for GH therapy occurred beyond this birth cohort and this group, by definition, was relatively old at the start of GH). For this analysis, we also excluded the last group born between 2000 and 2004 as their maximum age was only 11 years old in 2012 when KIGS data collection ended. Data included in statistical analysis of trends are shaded gray in Tables 2 and 3.

**Table 2:** Secular trends on birth parameters and growth (mean ± SD).

Birth year	Before 1980	1980–1984	1985–1989	1990–1994	1995–1999	2000–2004	*p*-Value
**At birth**							

*N* = [birth weight(BW)/birth length (BL)]	769/492	1163/777	1552/1123	1409/992	948/688	531/428	
BW SD score (SDS)	−1.18 ± 1.18	−1.11 ± 1.20	−1.04 ± 1.20	−1.00 ± 1.18	−0.99 ± 1.17	−1.00 ± 1.16	<0.001
BL SDS	–0.97 ± 1.42	−0.83 ± 1.38	−0.84 ± 1.41	−0.78 ± 1.38	−0.78 ± 1.53	−1.00 ± 1.37	<0.001
Midparental height SDS	−0.82 ± 1.17	−0.65 ± 1.17	−0.44 ± 1.14	−0.44 ± 1.16	−0.37 ± 1.17	−0.29 ± 1.16	<0.001

**At GHT start (cohort #1a)**							

*N* =	855	1306	1729	1621	1107	601	
Age (years)	12.68 ± 2.01	10.85 ± 2.93	9.70 ± 3.33	8.83 ± 3.44	7.43 ± 3.24	5.26 ± 2.39	<0.001
Height SDS (Prader)	−3.72 ± 1.05	−3.42 ± 1.02	−3.28 ± 0.95	−3.15 ± 0.98	−3.04 ± 1.22	−2.97 ± 1.13	<0.001
Height SDS (Ranke)	−0.05 ± 01.12	0.10 ± 1.10	0.21 ± 1.05	0.25 ± 1.07	0.30 ± 1.29	0.32 ± 1.08	<0.001
Δ Height − MPH SDS	−2.90 ± 1.11	−2.77 ± 1.18	−2.79 ± 1.16	−2.68 ± 1.19	−2.65 ± 1.37	−2.68 ± 1.23	<0.01
Weight SDS	−1.59 ± 1.31	−1.47 ± 1.32	−1.35 ± 1.30	−1.33 ± 1.36	−1.40 ± 1.40	−1.65 ± 1.38	NS
Body mass index (BMI) SDS	0.42 ± 1.08	0.37 ± 1.13	0.41 ± 1.16	0.39 ± 1.21	0.28 ± 1.17	−0.04 ± 1.21	NS

**At age 8 years (cohort #1b)**							

*N* =	18	226	287	280	178	102	
Height SDS (Prader)	−2.69 ± 0.79	−2.41 ± 0.79	−2.22 ± 0.77	−2.14 ± 0.78	−2.08 ± 0.86	−1.96 ± 0.87	<0.001
Height SDS (Ranke)	−0.44 ± 1.01	0.13 ± 1.00	0.10 ± 0.96	0.18 ± 0.98	0.27 ± 1.07	0.39 ± 1.09	<0.001
Δ Height − MPH SDS	−2.86 ± 1.06	−2.83 ± 1.11	−2.79 ± 1.16	−2.81 ± 1.14	−2.71 ± 1.12	−2.55 ± 1.32	NS
Weight SDS	−1.41 ± 1.32	−1.49 ± 1.15	−1.35 ± 1.20	−1.28 ± 1.24	−1.14 ± 1.25	−1.20 ± 1.15	NS
BMI SDS	0.63 ± 1.07	0.35 ± 0.97	0.35 ± 1.06	0.37 ± 1.13	0.48 ± 1.08	0.34 ± 0.95	NS

**Table 3:** Secular trends on pubertal timing (mean ± SD).

Birth year	Before 1980	1980–1984	1985–1989	1990–1994	1995–1999	2000–2004	*p*-Value
**At puberty start (cohort #2; all patients)**							

*N* = [birth weight(BW)/birth length (BL)]	401	536	652	487	136		
Age	14.39 ± 2.09	13.44 ± 2.02	13.37 ± 1.62	13.00 ± 1.81	12.42 ± 1.29		<0.001
Height SD score (SDS) (Prader)	−2.34 ± 0.98	−1.86 ± 1.04	−1.57 ± 0.92	−1.44 ± 0.90	−1.25 ± 0.99		<0.001
Height SDS (Ranke)	0.68 ± 1.21	1.28 ± 1.29	1.62 ± 1.19	1.81 ± 1.16	2.06 ± 1.30		<0.001
Δ Height − MPH SDS	−1.72 ± 1.09	−1.39 ± 1.17	−1.15 ± 1.14	−1.07 ± 1.12	−0.95 ± 1.02		<0.001
Weight SDS	−1.48 ± 1.32	−1.04 ± 1.40	−0.72 ± 1.27	−0.57 ± 1.32	−0.37 ± 1.19		<0.001
Body mass index (BMI) SDS	0.30 ± 1.08	0.38 ± 1.14	0.54 ± 1.14	0.60 ± 1.15	0.65 ± 1.02		<0.001
Age at menarche	16.05 ± 1.78	15.07 ± 1.79	14.82 ± 1.64	14.27 ± 1.85	13.86 ± 1.24		<0.001
Duration B2 to M1 (years)	1.82 ± 1.57	1.79 ± 1.35	1.93 ± 1.40	1.68 ± 1.68	1.97 ± 2.27		NS
Spontaneous puberty (%)	15	17	22	27	30		<0.001
Karyotype 45, *X* (%)	52	52	48	46	59		NS

**At puberty start (cohort #2; patients with induced puberty)**							

*N* =	340	445	509	323	95		
Age	14.38 ± 2.05	13.57 ± 1.94	13.66 ± 1.52	13.17 ± 1.46	12.68 ± 1.12		<0.001
Height SDS (Prader)	−2.34 ± 1.01	−1.82 ± 0.99	−1.52 ± 0.95	−1.36 ± 0.86	−1.10 ± 0.95		<0.001
Height SDS (Ranke)	0.69 ± 1.24	1.31 ± 1.24	1.69 ± 1.22	1.87 ± 1.16	2.24 ± 1.26		<0.001
Δ Height − MPH SDS	−1.80 ± 1.06	−1.37 ± 1.10	−1.18 ± 1.12	−1.06 ± 1.09	−0.92 ± 1.02		<0.001
Weight SDS	−1.44 ± 1.32	−1.07 ± 1.37	−0.72 ± 1.30	−0.57 ± 1.27	−0.35 ± 1.19		<0.001
BMI SDS	0.35 ± 1.05	0.35 ± 1.13	0.55 ± 1.14	0.61 ± 1.10	0.62 ± 1.02		0.003
Age at menarche	16.18 ± 1.71	15.33 ± 1.78	15.23 ± 1.55	14.61 ± 1.76	14.34 ± 0.87		<0.001
Duration B2 to M1 (years)	1.75 ± 1.53	1.76 ± 1.34	1.97 ± 1.52	1.52 ± 1.90	2.27 ± 2.65		NS

**At puberty start (cohort #2; patients with spontaneous puberty)**							

*N* =	61	91	143	119	41		
Age	14.45 ± 2.32	12.79 ± 2.31	12.37 ± 1.58	11.63 ± 1.64	11.80 ± 1.46		<0.001
Height SDS (Prader)	−2.36 ± 0.81	−2.04 ± 1.27	−1.73 ± 0.77	−1.47 ± 0.93	−1.59 ± 1.03		<0.001
Height SDS (Ranke)	0.63 ± 1.00	1.11 ± 1.48	1.40 ± 1.04	1.78 ± 1.20	1.63 ± 1.33		<0.001
Δ Height − MPH SDS	−1.29 ± 1.14	−1.47 ± 1.48	−1.06 ± 1.21	−0.93 ± 1.18	−1.04 ± 1.06		0.01
Weight SDS	−1.71 ± 1.29	−0.92 ± 1.54	−0.73 ± 1.18	−0.37 ± 1.34	−0.42 ± 1.19		0.008
BMI SDS	−0.00 ± 1.22	0.49 ± 1.22	0.50 ± 1.17	0.68 ± 1.24	0.72 ± 1.03		NS
Age at menarche	15.43 ± 2.04	13.76 ± 1.20	13.55 ± 1.23	13.10 ± 1.47	12.77 ± 1.31		<0.001
Duration B2 to M1 (years)	2.19 ± 1.75	1.89 ± 1.38	1.81 ± 0.98	2.05 ± 1.23	1.36 ± 1.08		NS

We divided data in three categories:

(a) Displayed and tested (presented as shaded data in Tables 2 and 3).
(b) Displayed but not tested (Ht and age), since these data are relevant where sufficient data are available but are likely prone to bias. Thus, data are displayed as they are still informative but are not tested.
(c) Not displayed and not tested (puberty), since only insufficient data are available.

Since age at the start of GH treatment changed over time, we additionally compared subgroups from each cohort who had pretreatment measurements at a comparable age of 8 years (between 7.0 and 9.0 years; cohort 1b). Mean exact age at this measurement did not differ across the birth year groups.

Cohort 2 was used to assess trends in puberty timing. It included only those TS subjects with data during the age period when puberty was expected to occur. Therefore, we excluded the last group born between 2000 and 2004 as their maximum age was only 11 years old in 2012 when KIGS data collection ended.

### Auxological Methods

Height was converted to SDS using both the height reference for healthy children of Prader (20) and the reference for TS of Ranke et al. (21). To calculate weight SDS, the normal population reference of Freeman et al. was used (22). To calculate body mass index SDS, the normal population reference of Cole was used (23). BW and BL for gestational age SDS were calculated using the reference of Niklasson et al. (24). The midparental height SDS was calculated as follows: (father’s height SDS + mother’s height SDS)/1.61 (25).

### Definitions

The onset of puberty was defined by the visit at which either spontaneous breast development (Tanner stage > B1) was first observed or the date at which estrogen replacement therapy was initiated. The assessment of the qualitative and quantitative aspects of estrogen replacement was done by the treating physicians. Furthermore, available data were stratified into whether pubertal development started spontaneously or was pharmacologically induced. The group with spontaneous start of puberty included patients with spontaneous progression of puberty until menarche as well as those who later required sex steroid substitution before menarche.

### Statistical Analysis

Statistical analyses [descriptive data analysis, calculation of SDS, and analysis of variance (ANOVA)] were carried out using SAS software (SAS Version 9.2, SAS Institute, Cary NC, USA). ANOVA models, *F*-tests, were applied to determine if there are any statistical mean differences between the groups based on year of birth. A *p*-value < 0.05 was considered to indicate statistical significance.

## Results

### Birth Parameters, Auxological Development, and GH Treatment

Distribution of BW and BL of all TS patients in whom birth parameters were available (*n* = 6372) are described in Table 1. Throughout the birth year cohorts “before 1980” until “1990–1994”, we observed a small ST for BW SDS with subsequent stabilization, with an increase of 0.18 SD over time, corresponding to about 157–180 g (depending on the gestational age; Table 2). In addition, a positive ST was observed in midparental height SDS.

**Figure 1:**
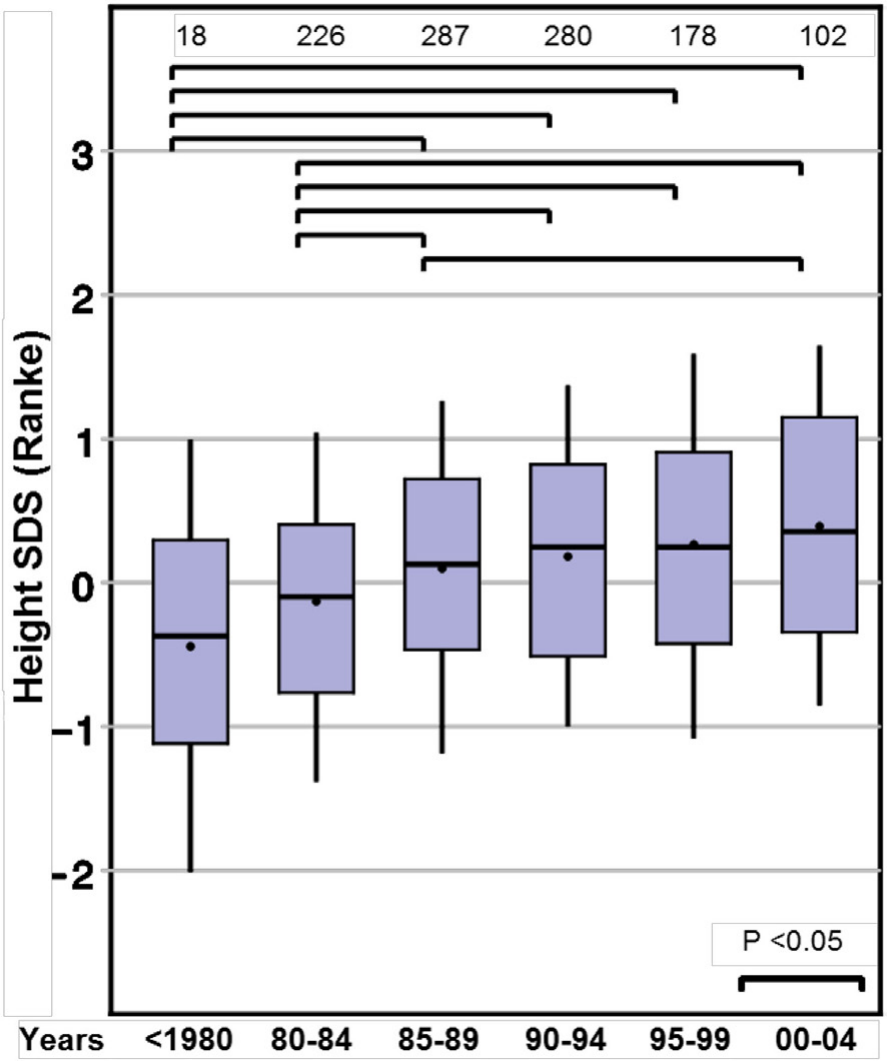
Secular trends on height in 8-year-old girls with Turner syndrome, height SD score (Ranke).

Height SDS at the start of GH therapy at the standardized age of 8 years (range between 7.0 and 9.0 years, see Methods) showed a positive ST between before 1980 and 2000–2004, for both Prader and Ranke height SDS statistics (Table 2) (Figure 1 for Ranke height SDS results). In addition, positive STs for height SDS (Prader and Ranke height SDS statistics) could also be observed both at the start of GH therapy (Table 2) and at the start of puberty (thelarche) (Table 3). Comparable to the ST in height SDS at 8 years of age, an ST in midparental height was also observed (+0.5 SD).

Age at the start of GH therapy declined substantially, from 10.8 years in the birth cohort 1980–1984 to 7.4 years in the birth cohort from 1995 to 1999. As described in Section “Methods”, the even more extreme mean ages at the start of GH in the before 1980 and 2000–2004 cohorts are likely artifactual, due to selection biases.

### Pubertal Development

Age at the start of puberty (whether induced or spontaneous) declined from 14.4 years in the before 1980 birth cohort to 12.4 years in the 1995–1999 cohort (*p* < 0.001). When stratified by spontaneous or induced puberty, STs toward an earlier start of puberty were evident in both subgroups, and the proportion with spontaneous puberty onset increased from 15% in those born before 1980 to 30% in those born 1995–1999 (Table 3).

To determine whether age at spontaneous puberty and age at the start of GH in patients with TS are associated, we performed a correlation analysis, revealing a highly significant correlation between age at the start of GH treatment and age at pubertal onset [correlation coefficient 0.65 (*p*-value < 0.0001)], which explained 32% of the variability in spontaneous puberty with age at GH start [intercept = 10.3 years (*p* < 0.0001), slope = 0.31 (*p* < 0.0001)].

**Figure 2:**
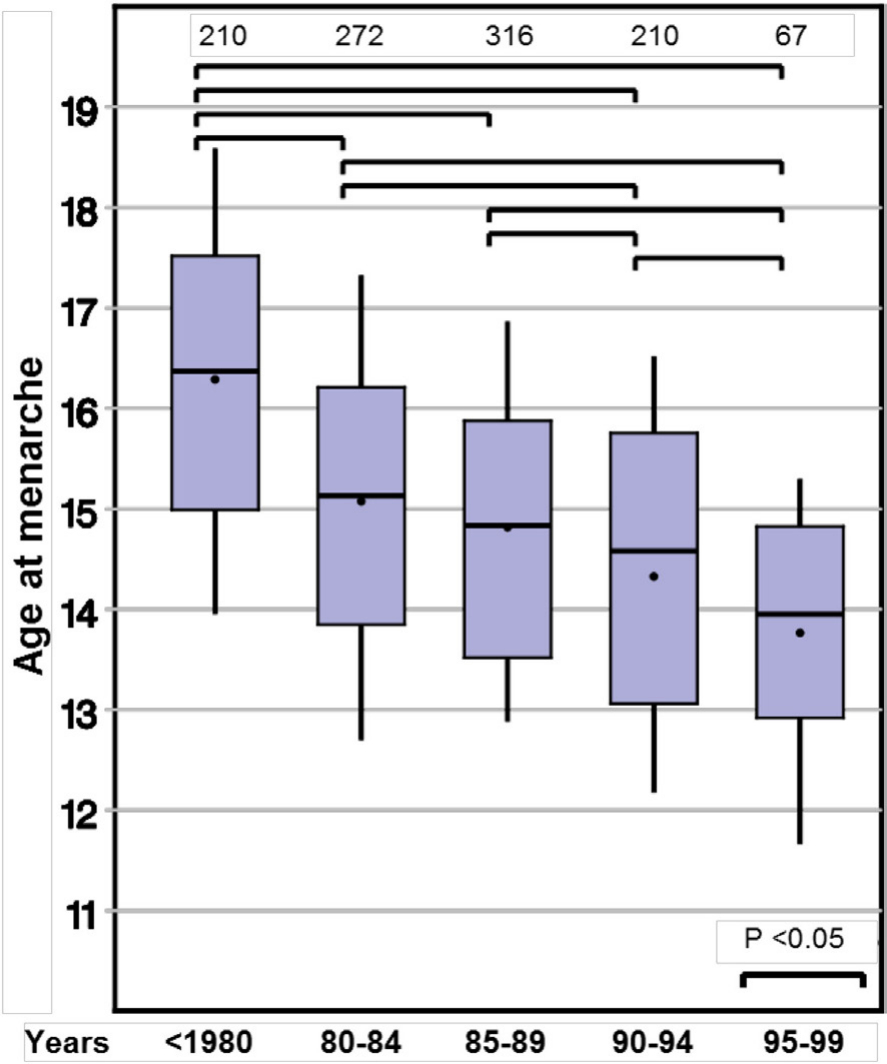
Secular trends on age at menarche in girls with Turner syndrome.

Age at menarche also declined substantially from 16.0 to 13.9 years (*p* < 0.001). Again, this observation remained significant in the two subgroups with either spontaneous or induced puberty (Table 3; Figure 2).

## Discussion

To the best of our knowledge, this is the first study analyzing whether TS girls display the same STs for auxological and pubertal development as those observed in the normal population. We found a highly significant ST for height at 8 years of age (+0.7 SDS). In addition, STs for height were present at the start of GH treatment and at the start of puberty. However, for these latter time points, the mean age differed greatly between the birth year groups, limiting direct comparisons. At the age of 8 years—before either the start of sex steroids or GH treatment may have influenced growth—the TS girls born after 2004 were more than half an SD taller compared to those born before 1980. Reported STs in height in normal populations differ between countries. Eastern and developing countries still show marked positive STs (26, 27), while conversely Western countries show only small (28) or even negative STs in growth (29). In contrast, our large group of TS girls included a wide variety of ethnicities, yet the positive ST in height SDS at the age of 8 years was observed for all, irrespective from their country of residence (data not shown). The remarkable positive ST of more than half an SDS, which corresponds to about 3 cm gain in height even without any endocrine treatment, surpasses contemporary estimates in the normal population. A height gain of 0.5 cm/decade or less would be expected for the corresponding birth year cohorts in most Western countries (30). Thus, the TS girls in this study born between 1975 and 2004 exhibited the same degree of ST on height at age 8 years as was observed one generation earlier in their parents.

It is important to notice that this positive ST in height could result in a delay of diagnosis because TS patients at the age of eight are nowadays nearly in the normal range or “less short.” However, our data show a constant shift toward an earlier start in GH treatment (reflecting probably the age of diagnosis) between the before 1980 and the post-2000 birth cohorts. Thus, although still a significant number of TS subjects seem to be diagnosed late, the awareness of TS seems to have improved despite less apparent phenotypical features.

We found a small positive ST for BW (+0.18 SD), but only a minor variation in BL. These findings are in line with data on healthy term infants who show a ST only for BW but not for BL. Comparison of recent growth curves and birth parameters (1, 24, 31) with historical data (2) showed a higher average BW for infants born at term. In contrast to that, BL remained constant in almost all the industrialized Western countries, and no change in this variable has been detected over the last 40 years. Higher maternal body weight and pregnancy weight gain (32) in recent years may be a reason for the observed gain in BW. Since both maternal and paternal genes contribute to infants’ birth parameters, the observed ST in midparental height might contribute to the observed changes in birth parameters. Furthermore, the ST in maternal height might be associated with alterations of the intrauterine environment, which again could be linked to the observed ST in birth parameters.

In recent years, epidemiological data from USA (33) and Denmark (8) showed STs toward earlier start of puberty in girls. We therefore were interested whether the same trend could be detected in TS patients in whom spontaneous puberty can be observed in about one third. We found a comparable decline in the age at spontaneous thelarche of about 2 years between those born before 1980 to those born in 2000–2004. As the time interval between visits was usually 6 months, the correct age at thelarche was likely earlier than that which we recorded. However, this limitation applied similarly for each of the five birth year groups with no expectation of bias.

The reasons behind the earlier spontaneous thelarche in TS subjects are unclear. We initially hypothesized that a lower threshold to karyotyping and a broader access to modern genetic diagnostics might have led to an increased proportion of patients with mosaicism, thereby explaining the doubling in the prevalence of spontaneous puberty and the decline in age at thelarche. However, as depicted in Table 3, the prevalence of patients with monosomy X did not change across the birth year groups. As in healthy girls, one can speculate that the increase in weight SDS of about one SD in the TS girls with spontaneous thelarche might have influenced the age at thelarche. However, in other settings the increase in weight did not fully explain the ST on puberty in healthy Danish girls (8, 34). Aksglaede and coworkers suggested that factors other than weight, such as changes in living conditions, nutrition during fetal development and childhood, and the wide distribution of endocrine disrupting chemicals (EDCs) (8, 35) might provoke the observed secular change. Probably, TS girls are exposed to the same changes in environmental conditions. Therefore, if EDCs are really causally related to the reported pubertal changes, these might also be related to the even more pronounced decrease in thelarche in TS girls.

As already reported in patients with idiopathic GH deficiency, age at puberty start correlated with age at the start of GH treatment in our study (36). Although the highly significant correlation between age at commencement of GH treatment and onset of spontaneous puberty is only an association and not proof of a causal relation, one can speculate that exposure to elevated GH concentrations might affect gonadotroph function, either through a direct or indirect effect (e.g., mediated by IGF-I). In this context, several *in vitro* studies have demonstrated that IGF-I is able to directly stimulate gonadotropin synthesis and secretion (37), so that a GH-induced increase in circulating IGF-I levels might contribute to the observed decline in age at the start of puberty. However, in a previous study in Italian TS patients, neither age at start nor prevalence of spontaneous puberty differed between GH-treated patients and a small non-GH treated control group who received androgen treatment (18). Furthermore, in our study, age at the start of GH treatment explains only 32% of the observed variability in spontaneous puberty, indicating that additional factors are probably involved in the physiology of earlier age at start and increased prevalence of spontaneous puberty.

The age at pharmacological induction of puberty decreased comparably to the age at spontaneous start of puberty. This phenomenon might be explained in particular by two factors: first, the decrease in age at the start of GH therapy (and probably age at diagnosis) might allow puberty induction at a more physiological age range. Second, the awareness of physicians for the psychosocial and physical sequelae of delayed puberty induction might have improved over time. In this context, one study has reported an even more improved height outcome for the early use of very low-dose estrogens in TS girls (38), and another study reported no significant influence of early start of low-dose estrogens on height development in TS girls (39). Together with the negative impact on bone health due to late estrogen exposure, these data argue strongly against a delay and in favor of an earlier starting age of puberty induction.

Although a ST in thelarche is found in several populations, all recent epidemiological studies (40) showed that the timing of menarche remained mostly unchanged with a consecutively longer interval between thelarche to menarche. In this study, we observed a significant reduction in age at menarche, both in TS girls with spontaneous as well as in those induced puberty. Whereas in TS girls with induced puberty probably the same explanations as for earlier age at thelarche might lead to the decrease in age at menarche, the reasons for the decrease in age at menarche in TS girls with spontaneous puberty remain unclear. Since the group with spontaneous start of puberty included subjects with spontaneous start but later requirement of sex steroid substitution before menarche, we speculate that the earlier menarche in this group is related to earlier sex steroid replacement therapy, comparable to the earlier start of pharmacologically induced puberty.

A major strength of this study is the unprecedented large study sample of girls with TS, allowing a comparison of growth and pubertal development over time in still sufficiently sized birth year cohorts. However, data from post-marketing studies such as KIGS have some important shortcomings. Therefore, the database contains no data from untreated TS subjects, which would allow determining whether the STs at birth, age 8 years or at the start of GH treatment translate to differences in adult height. Furthermore, several authors have speculated on the influence of socioeconomic factors as causative factors for the STs on height, which are not available in the database. Interobserver differences in a multicenter database in determining height and pubertal status are a potential weakness, but are probably balanced out by the large cohort size.

In summary, we find that trends toward increased childhood height and earlier pubertal onset operate not only in normal populations, but also in TS subjects, who also showed a doubling in the prevalence in spontaneous puberty onset between before 1980 to 1995–1999. In addition to these environment-related trends, awareness for TS seems to have improved, leading to earlier ages at the start of GH and pharmacological induction of puberty.

## Ethics Statement

The patients studied had received recombinant GH (Genotropin^®^, Pfizer Inc.) as part of the pharmacoepidemiologic survey known as KIGS^®^ (Pfizer International Growth Database). KIGS was established in 1987 as a worldwide observational registry to monitor outcomes and safety of Genotropin (somatropin, Pfizer Inc., New York, NY, USA) treatment in children with short stature. The KIGS survey was conducted in accordance with the Declaration of Helsinki.

## Author’s Note

KIGS is sponsored by Pfizer Inc.

## Author Contributions

FA, BG, AL, and JW developed the study design. FA, CC-H, BG, KO, AL, and JW discussed the findings. JW and BG wrote the first draft of the paper. All the authors contributed to draft revisions and approved the final manuscript.

## Conflict of Interest Statement

JW received grant support from Pfizer and Ipsen, and also lecture honoraria from Pfizer, Novo Nordisk, and Ipsen. AL was and FA and CC-H are Pfizer full-time employees. KO received honoraria from Pfizer as a member of the KIGS steering committee. Hypothesis development, analysis, interpretation, and conclusion contained in this study are those of the authors’ alone.

## Abbreviations

BL, birth length; BMI, body mass index; BW, birth weight; GH, growth hormone; ST, secular trend; TS, Turner syndrome.
